# Chaotic Zeeman effect: a fractional diffusion-like approch

**DOI:** 10.1038/s41598-024-57011-3

**Published:** 2024-03-16

**Authors:** Octavian Postavaru, Mariana M. Stanescu

**Affiliations:** Center for Research and Training in Innovative Techniques of Applied Mathematics in Engineering, University Politehnica of Bucharest, Splaiul Independentei 313, Bucharest, 060042 Romania

**Keywords:** Optics and photonics, Optical physics

## Abstract

It is shown that the chaotic Zeeman effect of a quantum system can be formally viewed as a result of fractional calculus. The fractional calculation brings into the equations the angle $\theta$ formed between the internal and the external magnetic field applied to the atom. The further the fractional coefficient $\alpha$ is from the ordinary case corresponding to $\alpha =1$, the more important the chaotic effect is. The case corresponding to $\alpha =1$ does not depend on the angle $\theta$, obtaining the nonchaotic situation known in the literature. Non-Gaussian distributions correspond to non-stationary variables. Considering a Lorenzian type distribution, we can make a connection between the fractional formalism and random matrix theory. The connection validates the link between fractional calculus and chaos, and at the same time due to the $\theta$ angle, it gives the phenomenon a physical interpretation.

## Introduction

Fractional equations model anomalous diffusion in many systems. In the paper^1^, it is shown that the probability density function of tracer particles’ radial displacements is strongly non-Gaussian showing algebraic decaying tails, and with the help of the fractional diffusion model it can be reproduced the shape and space-time scaling of the probability. The paper^2^ studied front dynamics in reaction-diffusion systems where anomalous diffusion is due to asymmetric Levy flights. The approach consists of replacing the Laplacian diffusion operator by a fractional diffusion operator. Motivated by work on contaminant spreading in geological formations, in the paper^3^, the authors propose and investigate a fractional advection-diffusion equation describing the biased spreading packet. In the solution of the variable-order fractional diffusion equation, the authors of the paper^4^ identified a new advection term that causes ultraslow spatial aggregation of subdiffusive particles due to dominance over the standard advection and diffusion terms in the long-time limit. In^5^, the authors derived a fractional Fokker-Planck equation for subdiffusion in a general space- and time-dependent force field from power law waiting time continuous time random walks biased by Boltzmann weights. The experiment does not involve quantum mechanics, it involves liquid crystal dynamics, and thus the related but still unsolved problem of whether it is indeed a quantum problem.

**Figure 1:**
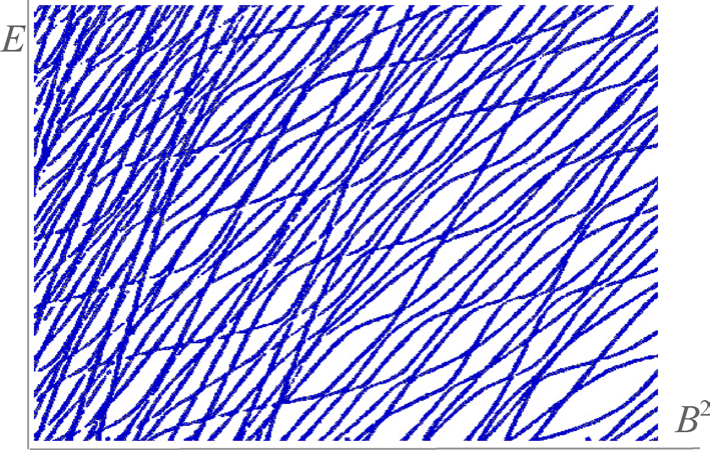
Chaotic distribution of energy levels: the spectra show irregular patterns that resemble well-stirred spaghetti.

The Zeeman effect is the consequence of placing an atom in a uniform external magnetic field, and results in the splitting of a spectral line into several components. The nature of the Zeeman splitting depends critically on the strength of the external magnetic field compared to the internal one. When the external magnetic field ${\textbf{B}}$ is small compared to the internal one ${\textbf{B}}_{\textrm{int}}$, the fine structure dominates and the problem is treated perturbatively. In the situation ${\textbf{B}}\gg {\textbf{B}}_{\textrm{int}}$ the Zeeman effect is dominant and the fine structure becomes perturbation. Also called the normal Zeeman effect, this phenomenon was first explained by Lorentz using Bohr’s atomic model. However, when the forces due to the external magnetic field become comparable to the Coulombian binding forces acting on the electron, the known theories break down and the energy levels become irregular, as we represented in Fig. 1. This transformation of the atomic spectrum from regular to irregular coincides with the situation in which the corresponding classical system transits from the regular to the chaotic regime. Next, we title this image of well-stirred spaghetti, chaotic^6^.

In the treatment of the non-chaotic Zeeman effect, we use the fact that due to the strength of the external magnetic field, the internal magnetic field is parallel to it, and the resulting theory does not depend on the angle $\theta$ that these two magnetic fields make between them. Inspired by the fact that the sequence of nearest level spacings has similarities with the diffusion process of a particle^7^, in this work, we propose an approach based on fractional calculus. Any deviation of the fractional coefficient $\alpha$ from $\alpha =1$, makes the theory depend on the angle $\theta$. In this paper, we show that this angle is responsible for the chaotic behavior of the process. We also note that random matrix theory predicts that the probability distribution of the distance between energy levels provides an excellent description of the energy levels in atoms excited in strong magnetic fields. We show that this is not accidental, by simply identifying the mean level density function with the Lorenzian profile we obtain an oscillating behavior similar to that given by the fractional calculus.

The Riemann-Liouville fractional integral is the inverse operator of the fractional derivative in the Caputo sense. Chaos is understood as an extreme sensitivity of the solutions of a system of differential equations to the initial conditions. Because Caputo derivatives share this sensitivity, they are a useful tool for extending regular behavior to irregular behavior. It is also known that chaos cannot appear in continuous systems with a number of equations less than three, except for fractional-order systems^8^. The Caputo derivative is also successfully used in optimal control problems to remove chaotic behaviors^9^. Last but not least, it should be mentioned that the Caputo derivative has the property of preserving the history of interactions in the dynamic behaviors it simulates, a characteristic property of singular kernel derivatives^10^.

The paper is structured as follows: in Section “Theoretical investigation” we conduct the theoretical investigation of the Zeeman effect by changing the definition of mechanical work from the Riemann integral to the Riemann–Liouville fractional integral. This allows us to link the energy levels of the atoms in the magnetic field to the $\theta$ angle. In Section “Results analysis”, we analyze the new behavior of the Zeeman effect and make the connection between fractional calculus and random matrix theory. We conclude the paper with the observation that the theory presented in this article can be successfully extended to any type of chaos, regardless of whether it is classical or quantum.

## Theoretical investigation

### Definition 1

^11^ Magnetic moment of the atom in a uniform magnetic field ${\varvec{\mu }}_l$ is a consequence of the fact that the electron performs an orbital movement around the nucleus, and is defined as

1 $$\begin{aligned} {\varvec{\mu }}_l=-\frac{e}{2m}{\textbf{L}}, \end{aligned}$$

where ${\textbf{L}}$ is the orbital angular momentum of the electron, *e* is the electron charge and *m* is the mass of the electron.

### Definition 2

^11^ When an atom is placed in a magnetic field ${\textbf{B}}$, the torque acting on it is defined

2 $$\begin{aligned} {\varvec{\tau }}_l={\varvec{\mu }}_l\times {\textbf{B}}=\mu _l B \sin \theta {\hat{e}}_{\tau }. \end{aligned}$$

It means that the magnetic field works on the dipole to rotate it.

### Definition 3

^11^ The mechanical work done by the field on the system will be stored in terms of potential energy, and it must correspond to the rotation of the dipole from $\pi /2$ to $\theta$

$$\begin{aligned} U(\theta )=\int _{\pi /2}^{\theta }\tau _l d\theta \equiv \left. I(\tau _l)\right| _{\pi /2}^{\theta }. \end{aligned}$$

### Definition 4

We make the following ansatz

3 $$\begin{aligned} U^{\alpha }(\theta )= \left. I^{\alpha }(\tau _l)\right| _{\pi /2}^{\theta }, \end{aligned}$$

where the Riemann-Liouville integral operator $I^{\alpha }$ is defined as^12^

$$\begin{aligned} I^{\alpha }f(t)=\frac{1}{\Gamma (\alpha )}\int _{0}^{t}(t-\tau )^{\alpha -1}f(\tau )d\tau . \end{aligned}$$

### Proposition 1

^13^*We have the following result*

$$\begin{aligned} I^{\alpha }(\sin \theta )=\frac{\theta ^{\alpha }}{2i}\left( E_{1,1+\alpha }(i\theta )-E_{1,1+\alpha }(-i\theta )\right) \equiv f(\theta ,\alpha ), \end{aligned}$$

where the two-parameter Mittag-Leffler function is given by the following expression^14^

$$\begin{aligned} E_{\alpha ,\beta }(z)=\sum _{k=0}^{\infty }\frac{z^k}{\Gamma (\alpha k+\beta )},\quad z\in {\mathbb {C}}. \end{aligned}$$

### Proposition 2

*Let *$\mu _B=e\hbar /(2m)$ *be the Bohr magneton, with e the electron charge and* $\hbar$ *the reduced Planck constant. Then we have*

$$\begin{aligned} U^{\alpha }(\theta )=\mu _Bm_lB\,F(\theta ,\alpha ), \end{aligned}$$

with

$$\begin{aligned} F(\theta ,\alpha )=-\frac{1}{\cos \theta }\left( f(\theta ,\alpha )-f\left( \frac{\pi }{2},\alpha \right) \right) . \end{aligned}$$

### *Proof*

Starting from the definition given by Eq. (3) and using Eq. (2), we obtain

$$\begin{aligned} U^{\alpha }(\theta )= & {} \mu _l B \left. I^{\alpha }(\sin \theta )\right| _{\pi /2}^{\theta }=\mu _l B \left. (f(\theta ,\alpha ))\right| _{\pi /2}^{\theta }=-{\varvec{\mu }}_l\cdot {\textbf{B}}\,F(\theta ,\alpha )\\= & {} \frac{e}{2m}{\textbf{L}}\cdot {\textbf{B}}\,F(\theta ,\alpha )=\frac{e}{2m}LB\cos \theta \,F(\theta ,\alpha )=\frac{e}{2m}(L\cos \theta ) B\,F(\theta ,\alpha )\\= & {} \frac{e}{2m}L_z B\,F(\theta ,\alpha ). \end{aligned}$$

Because $L_z=m_l\hbar$, we get the desired result. $\square$

### *Remark 1*

When $\alpha =1$, $F(\theta ,\alpha =1)=1$, regardless of $\theta$. This means that in the ordinary case, the potential energy does not depend on the angle between ${\textbf{L}}$ and ${\textbf{B}}$.

**Figure 2:**
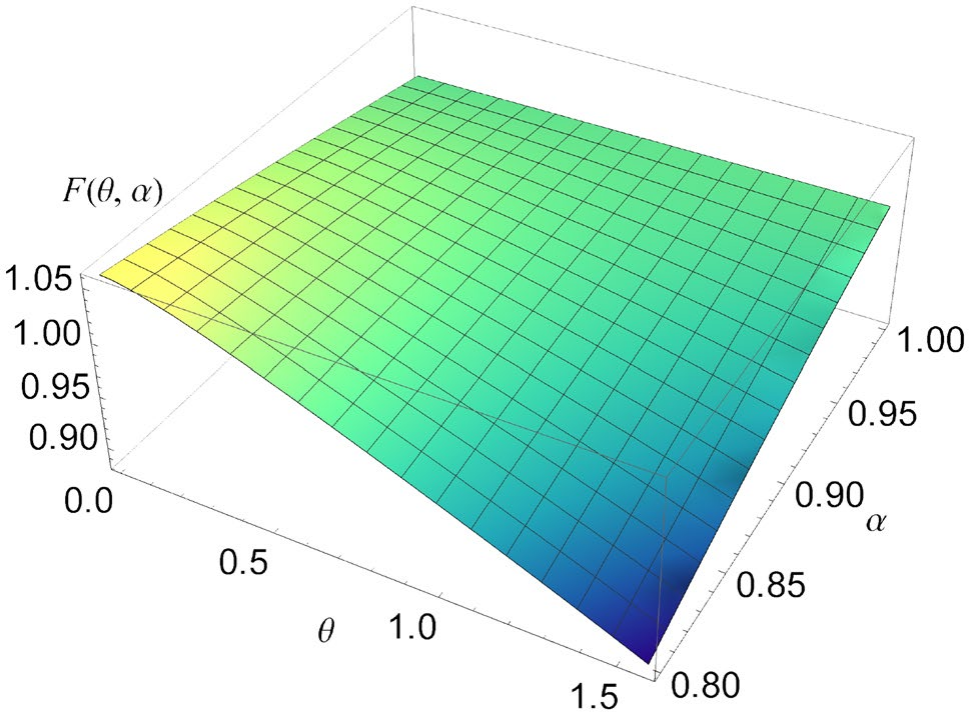
The function $F(\theta ,\alpha )$, for $\alpha \in [0.8,1]$ and $\theta \in [0,1.5]$. When $\alpha =1$, the function is independent of $\theta$.

### *Proof*

We start from the fact that

$$\begin{aligned} E_{1,2}(z)=\sum _{k=0}^{\infty }\frac{z^k}{\Gamma (k+2)}=\frac{1}{z}\sum _{k=0}^{\infty }\frac{z^{k+1}}{\Gamma (k+2)}=\frac{e^z}{z}, \end{aligned}$$

we get

$$\begin{aligned} f(\theta ,1)=-\frac{1}{2}\left( e^{i\theta }+e^{-i\theta }\right) =-\cos \theta , \end{aligned}$$

and as a consequence

$$\begin{aligned} F(\theta ,1)=\frac{1}{\cos \theta }\left( \cos \theta -\cos \frac{\pi }{2}\right) =1. \end{aligned}$$

$$\square $$

In Fig. 2, we represented the function $F(\theta ,\alpha )$, for different values of $\alpha$ and $\theta$. It can be seen that if $\alpha =1$, the function is not dependent on $\theta$ and is equal to 1.

### Definition 5

^11^ In the absence of the magnetic field, the jump of an electron between two energy levels $E_1$ and $E_2$ is accompanied by a photon with frequency

$$\begin{aligned} \nu _0=\frac{E_2-E_1}{h}, \end{aligned}$$

where $E_2$ is the excited energy.

### Definition 6

If the atom is placed in a magnetic field then the total energy $E_t$ of the atom is

4 $$\begin{aligned} E_t=E+U^{\alpha }(\theta )=E+\mu _Bm_lB\,F(\theta ,\alpha ). \end{aligned}$$

**Figure 3:**
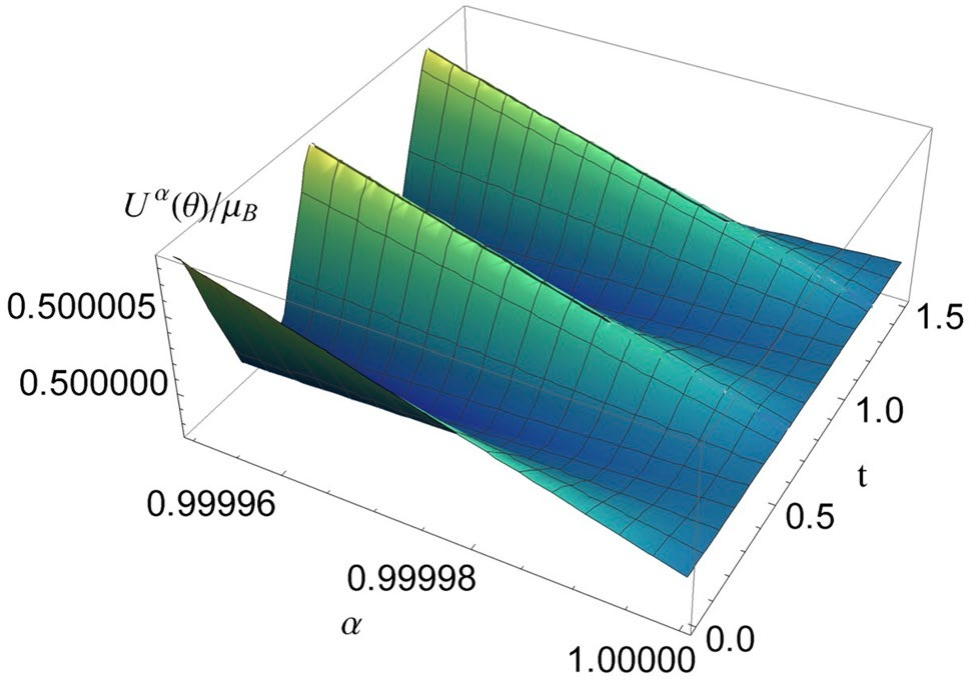
The function $U^{\alpha }(\theta )/\mu _B$, with $\theta (t)=\sin 5t$ for $\alpha \in [0.99995,1]$, $t\in [0,1.5]$ s, and $B=0.5$ T. Moving $\alpha$ away from 1 increases the chaotic behavior.

### Proposition 3

For an atom in a magnetic field, the transition of an electron is accompanied by a photon of frequency

5 $$\begin{aligned} \nu =\nu _0+\Delta m_l\frac{e}{4\pi m}B\,F(\theta ,\alpha ), \end{aligned}$$

with $$\Delta m_l=m_l^2-m_l^1$$.

### *Proof*

According to Eq. (4), the energies corresponding to the two energy levels involved are

$$\begin{aligned} E_{t2}=E_2+m_l^2\mu _BB\,F(\theta ,\alpha ),\quad E_{t1}=E_1+m_l^1\mu _BB\,F(\theta ,\alpha ), \end{aligned}$$

and therefore

$$\begin{aligned} \nu =\frac{E_{t2}-E_{t1}}{h}=\nu _0+(m_l^2-m_l^1)\frac{e}{4\pi m}B\,F(\theta ,\alpha ). \end{aligned}$$

$$\square $$

### *Remark 2*

We may face the following situations:

(i) ($$\pi $$ line) $$\Delta m_l=0$$ $$\rightarrow $$ $$\nu =\nu _0$$,
(ii) $$\Delta m_l=1$$ $$\rightarrow $$ $$\nu =\nu _0+\tfrac{e}{4\pi m}B\,F(\theta ,\alpha )$$,
(iii) $$\Delta m_l=-1$$ $$\rightarrow $$ $$\nu =\nu _0-\tfrac{e}{4\pi m}B\,F(\theta ,\alpha )$$.

This means that in the situation where $\Delta m_l=0$, chaos has no contribution.

### *Remark 3*

When $\alpha =1$, we have $F(\theta ,\alpha )=1$ and $U(\theta )=\mu _Bm_lB$, a result that allows us to obtain the ordinary situation from the literature^11^

$$\begin{aligned} \nu =\nu _0+\Delta m_l\frac{e}{4\pi m}B. \end{aligned}$$

## Results analysis

We are interested in analyzing the behavior of Eq. (4), more precisely the dependence of $U^{\alpha }(\theta )$ according to the magnetic field *B* and the function $F(\theta ,\alpha )$, knowing that $m_l=1$ and that $\mu _B=5.788\times 10^{-5}$ eV T^-1^.

The angle $\theta$ will oscillate around 0, and for a value description we assume that we have the following variation of $\theta$ with the time $\theta (t)=\sin 5t$. In Fig. 3 we represented $U^{\alpha }(\theta )/\mu _B$, and as we can see, by how much $\alpha$ departs from the value 1, the oscillation depending on $\theta$ becomes stronger. So the value of $\alpha$ is closely related to the deviation of the energy spectrum from the ordinary behavior. The farther $\alpha$ has a value from 1, the more prominent the chaotic behavior.

**Figure 4:**
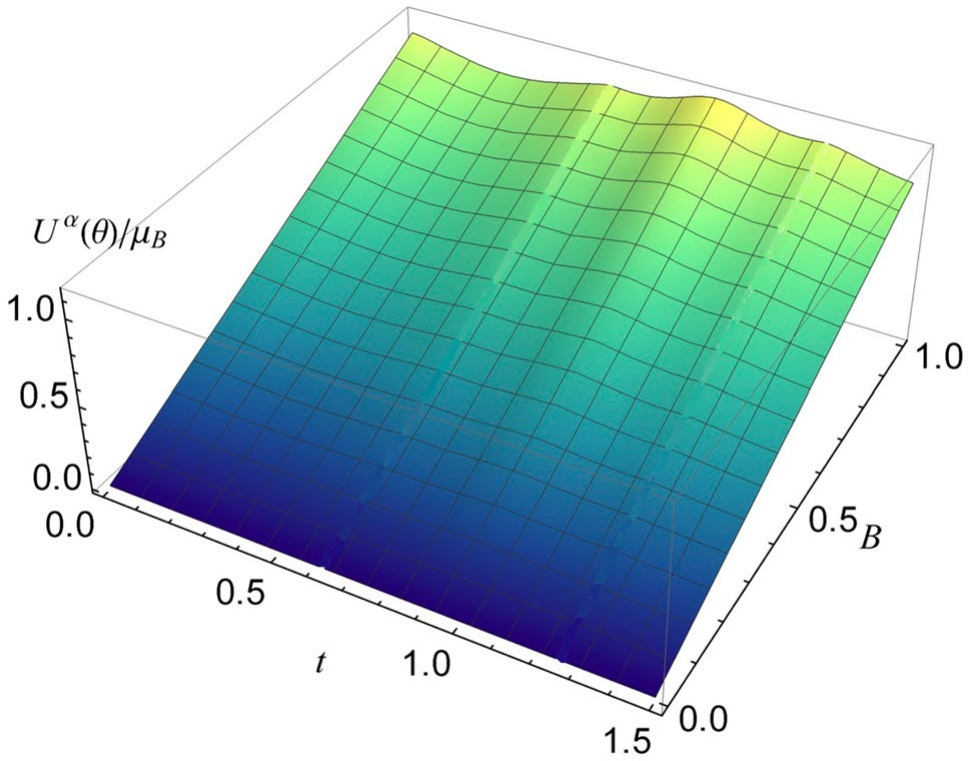
Irregular patterns of $U^{\alpha }(\theta )/\mu _B$ for $\alpha =0.8$, $B\in [0,1]$ T, and $t\in [0,1.5]$ s.

Fixing the value of $\alpha =0.8$, In Fig. 4 we analyze the behavior of $U^{\alpha }(\theta )/\mu _B$ depending on the magnetic field *B* and the angle $\theta$ considering the same time dependence as in Fig. 2. This tells us that for a given value of *B*, the function can have any of the values given by $t\in [0,1.5]$ s. In this way, a spectrum is obtained that shows irregular patterns that resemble well stirred spaghetti^6^.

Random matrix theory provides an excelent description of the spacing of the measured energy levels in excited nuclei. Although there are no random elements in atomic phenomena, the statistics of the energy-level spacing agreed remarkably well with the prediction of the random matrix theory. The unfolded enegy levels are defined^7^

6 $$\begin{aligned} {\overline{E}}(\beta ,\epsilon )=\int _{-\infty }^{\epsilon }{\overline{\rho }}(\beta ,E')dE', \end{aligned}$$

where ${\overline{\rho }}(\beta ,E')$ is the mean level density function, and $\beta$ stands for some parameters defining the functional form of $\rho$.

We notice that Eq. (4) comes from an integral over time, and Eq. (6) is described by an integral over energy. If there is a connection between the two functions, this is given by the Fourier transform.

Non-Gaussian distributed data arise when the mean statistics vary with time, which means that the variable could be non-stationary: time-varying (its trend changes with time), or cross-correlated (it changes depending on its previous value or the values of other variables). Since the fractional calculation takes into account the history of the process, we think of using a non-Gaussian distribution. We choose a Lorentzian distribution for the unfolded energy levels, i.e.,

$$\begin{aligned} {\overline{E}}(E_0,\Gamma ,\epsilon )=\frac{1}{\pi }\frac{\Gamma /2}{(\epsilon -E_0)^2+(\Gamma /2)^2}, \end{aligned}$$

and then its Fourier transform has the expression

$$\begin{aligned} {\mathscr {F}}\left[ {\overline{E}}(E_0,\Gamma ,\epsilon )\right] (t)=e^{-2\pi i t E_0-\Gamma \pi \vert t\vert }, \end{aligned}$$

and the real part of this function when $\Gamma \rightarrow 0$ is a sinusoidal. This behavior is similar to that represented in Fig. 3, for a given value of $\alpha$. The fact that a link can be created between the two formalisms once again justifies the usefulness of fractional calculus in describing chaotic behaviors.

## Conclusions and outlook

Inspired by the description of diffusion, we used fractional calculus to describe the chaotic Zeeman effect. Fractional calculus has recently been used to model various phenomena. In the paper^15^, the authors studyed the existence of numerical solution and stability of a chemostat model under fractal-fractional order derivative. The analysis proved to be an excellent tool for discussing the fractal characteristics of porous media. The manuscript^16^ focused on the modeling and numerical solution of the dynamical model of typhoid fever. The modeling was based on the Atangana-Baleanu operator with the Mittag-Leffler function in Caputo sense. In the article^17^, the authors studyed the Drinfeld-Sokolov-Wilson equation considered in fractal-fractional sense with exponential decay and Mittag-Leffler type kernels. The dynamics of COVID-19 is investigated in^18^, where the authors used a fractional order SEIR model.

Compared to random matrix theory, in which we do not have random elements in atomic physics, in our theory the chaos depends on the fluctuation of the $\theta$ angle between the intrinsic magnetic moment and the magnetic field applied to the atom. Figure 3 shows us that when $\alpha =1$, case corresponding to the non-chaotic Zeeman effect, the energy levels do not depend on the $\theta$ angle. Eq. (5) represents the chaotic unfolding of the energy spectrum. As can be seen in Fig. 3, the fluctuation amplitudes of the energy levels are modulated by the fractional value of the coefficient $\alpha$. In Fig. 4, you can see the irregular spectrum reminiscent of well stirred spaghetti.

Since the fractional calculation introduces the history of the process into the dynamics, we choose to use a non-Gaussian distribution for the data. The Fourier transform of the Lorentz distribution has a sinusoidal shape, which makes the connection with the oscillating behavior described in Fig. 3. The link between the two formalisms justifies the effectiveness of fractional calculus in describing chaotic processes. At the same time, due to the physical interpretation of the angle $\theta$, the formalism described by us gets a physical interpretation.

We expect the oscillation frequency of ${\textbf{L}}$ around ${\textbf{B}}$ to be high, or in other words $\theta$ to oscillate rapidly around the value $\theta =1$, value corresponding to the non-chaotic energy spectrum. This property is known as rigidity of the energy spectrum. This effect is similar to the vibrations obtained in high voltage wires. Analog^7^, we can consider the energy spectrum as a discrete signal and the sequence of energy levels as a time series. In fact, we proceeded similarly in Fig. 3, where we generated a time series when we considered $\theta (t)=\sin 5t$. In a time series, antipersistence means that an increasing or decreasing trend in the past makes the opposite trend in the future, and quantum systems are very antipersistent (characterized by 1/*f* noise). The rigidity of the spectrum as well as its rapid oscillation around the non-chaotic value obtained with our theory, are consistent with the antipersistent cataloging from^7^.

Turbulence is easily associated with fractals, and fractals represent the geometry of chaos. Probably the best known example of turbulence is the vortex on Jupiter. Looking closer and closer, we see first larger vortices, then smaller ones, keeping the structure of the whole on a reduced scale. Geometric objects with this structure are called fractals and have a different geometry than ordinary geometry. For example, a sphere viewed more and more closely becomes more and more flat. Fractals are different, when we enlarge them we see more and more structure.

An idea to study the Zeeman effect from this point of view is to modify Eq. (3), replacing the Riemann-Liouville integral operator with an fractal or fractal-fractional integral operator. In the literature there are several operators of this type^19,20^. Maybe this type of operators bring out a new dynamic of the electronic spectrum highlighting aspects still undiscovered related to the Zeeman effect.

In fact, the analysis in this paper, although applied to quantum chaos, should work successfully in any kind of chaos, regardless of whether it is classical or quantum.

## Data Availability

All data generated or analysed during this study are included in this published article.
